# 

**Published:** 1914-11

**Authors:** 


					﻿OFFICIAL ORGAN—State Society Social Hygiene aim Eugenics.
VoL XXX
NOVIEMBER, 1914
No. 5
■“Red Back
TEXAS MEDICAL JOURNAL
■
IS
in
st
H
H
SH
a
MM
E
h
ESTABLISHED JULY. 1885
PUBLISHED MONMlYATAUSTIN, ATA BY
MRI . F. E*. DANIEL, - p - Publisher annOgma^dj 0TifcP^
PRNTTDBYTTS.TONEVECKMANOJoCES Ma
SbrsernptOn)o,S HOO alinTinAdn’anee. s. .	^Vi-SgseiCOpSesrlW-C^*
Enterrdallh0OhUofflcertAuutnn,T-xas, asusonSelT rxUraaUer.
				

